# ZIF-67 In Situ Grown on Attapulgite: A Flame Retardant Synergist for Ethylene Vinyl Acetate/Magnesium Hydroxide Composites

**DOI:** 10.3390/polym14204408

**Published:** 2022-10-19

**Authors:** De-Xin Ma, Yuan Yang, Guang-Zhong Yin, Antonio Vázquez-López, Yan Jiang, Na Wang, De-Yi Wang

**Affiliations:** 1 Liaoning Provincial Key Laboratory for Synthesis and Preparation of Special Functional Materials, Shenyang University of Chemical Technology, Shenyang 110142, China; 2 Escuela Politécnica Superior, Universidad Francisco de Vitoria, Ctra. Pozuelo-Majadahonda Km 1800, 28223 Madrid, Spain; 3 IMDEA Materials Institute, C/Eric Kandel, 2, Getafe, 28906 Madrid, Spain; 4 Shenyang Research Institute of Industrial Technology for Advanced Coating Materials, Shenyang 110142, China

**Keywords:** ZIF-67, attapulgite, magnesium hydroxide, EVA, flame retardant

## Abstract

ZIF-67@ATP was prepared by the in situ growth of the zeolite imidazole frame (ZIF-67) on the surface of attapulgite (ATP). The structure and surface morphology of ZIF-67@ATP were characterized by Fourier transform infrared spectroscopy (FTIR), X-ray diffraction (XRD), X-ray photoelectron spectroscopy (XPS), scanning electron microscopy (SEM) and transmission electron microscopy (TEM). Different mass fractions of ATP and ZIF-67@ATP were added to ethylene vinyl acetate (EVA)/magnesium hydroxide (MH) composites as flame retardant synergists. The flame retardancy of EVA composites was evaluated by the limiting oxygen index (LOI) test, UL-94 test and cone calorimeter test. Composites containing 3 wt% of ZIF-67@ATP reached an LOI value of 43% and a V-0 rating in the UL-94 test, and the ignition time of the composite increased from 38 s to 56 s. The tensile strength and impact strength of the composites did not change significantly, but the elongation at break increased greatly. Typically, for composites containing 4 wt% of ZIF-67@ATP, the elongation at break of the composites increased from 69.5% to 522.2% compared to the samples without the synergist. This study provides novel insights into the application of attapulgite in the field of flame retardant polymer materials.

## 1. Introduction

Ethylene vinyl acetate (EVA) can be used in various industrial fields depending on the different vinyl acetate content [1,2]. EVA has excellent insulation performance, so it is widely used in the wire and cable industry [3]. However, EVA is easy to burn, and the droplet is significant during combustion, seriously limiting the application of EVA. Adding a flame retardant to the EVA matrix is an effective method to improve the overall flame retardancy. Among different flame retardants, halogenated flame retardants are highly effective. However, with the increase in people’s awareness of environmental protection, the application of halogenated flame retardants is gradually decreasing [4,5]. Inorganic flame retardants such as aluminum hydroxide (ATH), magnesium hydroxide (MH) and layered double hydroxide (LDH) have attracted more and more attention because they are environmentally friendly [6,7,8]. Among them, MH has the advantage of being non-toxic and harmless and having a high decomposition temperature, low price and easy storage and transport [9]. However, MH has the drawbacks of low flame retardant efficiency and the need to use high content in the matrix of the host materials [10].

Common inorganic nanomaterials include montmorillonite [11], sepiolite [12] and attapulgite [13], among others. Attapulgite is a kind of natural clay, mainly composed of hydrated magnesium aluminosilicate [14]. Its microscopic morphology has a rod-like shape, which has less than 100 nanometers in diameter and hundreds of nanometers or several microns in length [15]. The surface of attapulgite has exchangeable cations and abundant hydroxyl groups, so it has been widely used in sewage treatment, catalytic carriers and as filler for plastics, rubber, coatings and other industrial products [16,17,18]. However, attapulgite (ATP) has strong hydrophilicity and poor interface interaction with polymer materials. Therefore, it is easy for attapulgite to agglomerate in the matrix of the host material. To use attapulgite effectively, it is very important to modify its surface and improve the compatibility between attapulgite and matrix materials. In recent years, many related studies have also been reported. Niu et al. [19] improved the dispersion of attapulgite by forming a covalent bond between attapulgite and epoxy resin through acrylic resin. When the content of acrylic acid-treated attapulgite reached 3 wt%, the storage modulus of epoxy resin reached 128.9%, and when it reached 5 wt%, Young’s modulus and tensile strength of epoxy resin increased by 75.5% and 188.3% compared with those of pure epoxy resin. Gao et al. [20] modified ATP by the free radical polymerization of acrylic acid and 3-methacryloxypropyl-trimethoxysilane to prepare polyacrylic acid/attapulgite (PA/ATP) nanocomposites. It was found that the thermal stability of the treated cotton fabric was improved, the limiting oxygen index (LOI) reached 24.7%, and the mechanical properties were slightly improved. Ju et al. [21] modified the surface of attapulgite with phosphorus flame retardant resorcinol bis (diphenyl phosphate) (RDP), prepared a new flame retardant called RDP-coated Nano ATP (R-NATP) and used it for the flame retardant modification of polylactic acid. When the addition of R-NATP reached 30%, the LOI of polylactic acid composites reached 24.5%, and the V-0 rating was obtained in the vertical combustion test.

Metal-organic frameworks (MOFs) are considered potential flame retardant substances with proper flame retardancy. There are increasing prospects for MOF-based composites to promote fire safety in polymers [22,23]. ZIF-67, which is composed of cobalt ions, is a widely studied subset of MOFs and has been widely used in various fields due to its attractive properties [24,25]. In situ growth of MOFs on the surface of inorganic materials is a novel method of inorganic material modification. Inorganic materials modified in this way are widely used in wastewater treatment, catalysis, energy storage materials and other fields [26,27]. However, it is hardly found that MOF in situ growth-modified inorganic filler is used in the field of polymer flame retardant modification. Therefore, in this work, the idea of in situ growth of MOFs on the surface of attapulgite and its application in flame retardant modification of EVA is developed. Herein, ZIF-67 is used to modify the surface of attapulgite, acting as a flame retardant synergist. Then, it is introduced into EVA/MH composites to form a synergistic flame retardant system with MH. The different effects of pure ATP and ZIF-67@ATP on the flame retardant and the mechanical properties of EVA/MH composites will be also discussed.

## 2. Experimental Section

### 2.1. Materials

Ethylene vinyl acetate (EVA, UL00628) was supplied by Shandong Zhongbo New Materials Co., Ltd. (Jinan, China); magnesium hydroxide (MH) was obtained from Liaoning Jinghua New Material Co., Ltd. (Haicheng, China); attapulgite came from Xuyi, Shandong Province; concentrated hydrochloric acid was supplied by Shenyang Xinhua Reagent Factory (Shenyang, China); Co(NO_3_)_2_·6H_2_O and ethanol were provided by Tianjin Damao Chemical Reagent Factory (Tianjin, China); 2-methylimidazole was purchased from Shanghai Macklin Biochemical Technology Co., Ltd. (Shanghai, China). All chemicals were of analytical grade purity and used without any further purification.

### 2.2. Pretreatment of Attapulgite

The surface of commercially available attapulgite presented some impurities and a variety of metal elements in low content. The existence of these impurities and metal elements will reduce the activity of hydroxyl groups on the surface of attapulgite. Therefore, it was necessary to activate the surface of attapulgite with concentrated hydrochloric acid. Thus, 10 g of commercially available attapulgite was added into 1 L of 8 mol/L concentrated hydrochloric acid solution, stirred at 70 °C for 12 h and then washed with deionized water until it reached neutrality. Then, the acid treatment product was collected by suction filtration and further dried in a 60 °C vacuum oven for 12 h. The obtained product was ground with a disintegrator for standby and named acid-treated ATP (a-ATP) [28].

### 2.3. Preparation of ZIF-67@ATP

**Scheme 1:**
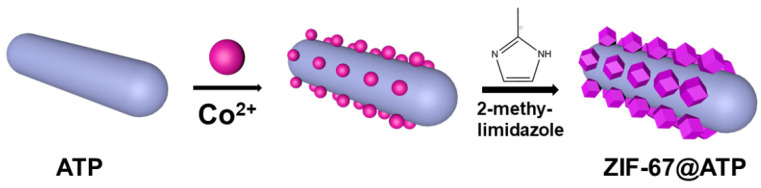
Preparation process of ZIF-67@ATP composites.

Next, 8.162 g (0.08 mol) of the a-ATP prepared in the previous step was dispersed into 300 mL ethanol and named solution A. Then, 5.82 g (0.02 mol) of Co(NO_3_)_2_·6H_2_O and 0.002 mol HCI were dispersed into 100 mL of ethanol, and the solution was named solution B. Solution A and solution B were mixed and subjected to ultrasonic treatment for 2 h. Next, 2-methylimidazole (16.4 g, 0.2 mol) was dissolved in 200 mL ethanol to prepare solution C. The above three solutions were mixed and stirred under ultrasonic bath at room temperature for 5 h. The reaction product was then washed with ethanol and deionized water and dried in a 60 °C vacuum oven for 12 h. The steps of the synthesis process are summarized in Scheme 1.

### 2.4. Fabrication of EVA/MH/ZIF-67@ATP Composites

EVA, MH and ZIF-67@ATP were prepared into composite materials according to the concentrations shown in Table 1 by using an open mixer. The temperature of the front roll and the back roll were both set to 80 °C.

### 2.5. Characterization

Fourier infrared spectroscopy (FTIR) spectra were obtained with a Nicolet magna-ir 560 spectrometer (FTIR, Nicolet MAGNA-IR560, Artisan Technology Group, Champaign, IL, USA), in the range of 400 cm^−1^ to 4000 cm^−1^. X-ray diffraction (XRD) was obtained with a Bruker D8 Advance diffractometer (Karlsruhe, Germany) working with Cu Kα radiation (0.15418 nm). Thermogravimetric analysis (TGA) results were obtained in the range from 40 °C to 700 °C using an STA 449C thermo analyzer instrument (Netzsch scientific instruments, Selb, Germany) with a heat ramp of 10 °C/min under nitrogen atmosphere. Transmission electron microscopy (TEM, Talos F200X, FEI, Thermo Fisher Scientific company, Waltham, MA, USA) was employed to observe the morphology of ZIF-67@ATP. Scanning electron microscope (SEM, Thermo Fisher Scientific company, Waltham, MA, USA) equipped with energy dispersive X-ray spectroscopy (EDX, inspection F50) was employed to observe the surface element distribution of ZIF-67@ATP and the morphology of carbon residue. Limit oxygen index (LOI) test was measured according to ASTM Standard D2863–13, with an SS-1005 oxygen index meter from Taiwan Songshu Testing Instrument company (Taiwan, China). The sample size was 130 mm × 6.5 mm × 3.2 mm. Vertical combustion test was performed according to ASTMD380 using a vertical burning tester (SS-3001, Taiwan Songshu Testing Instrument Company, Taiwan, China) with sample dimensions of 127.0 mm × 12.7 mm × 3.0 mm. Cone calorimeter test was performed according to ISO 5660-1 using a cone calorimeter tester (Fire Testing Technology Limited, West Sussex, UK) with sample size of 100.0 mm × 100.0 mm × 4.0 mm under a heat flux of 50 kW/m^2^. Tensile testing measurements were performed on an Electronic universal material testing machine (9250HV, Instron Engineering Corporation, Norwood, MA, USA) following ISO 37/2017, the average values of three samples in each group were taken. Impact strength test was conducted according to ISO 2932003 using an impact testing machine (Taiwan Gotech Testing Machines Inc., Taiwan, China) with sample size of 80 mm × 10 mm × 4 mm. The average value of five samples in each group was taken, and the impact span was 60 mm.

## 3. Results and Discussion

### 3.1. Structure and Morphology of ZIF-67@ATP Composites

**Figure 1:**
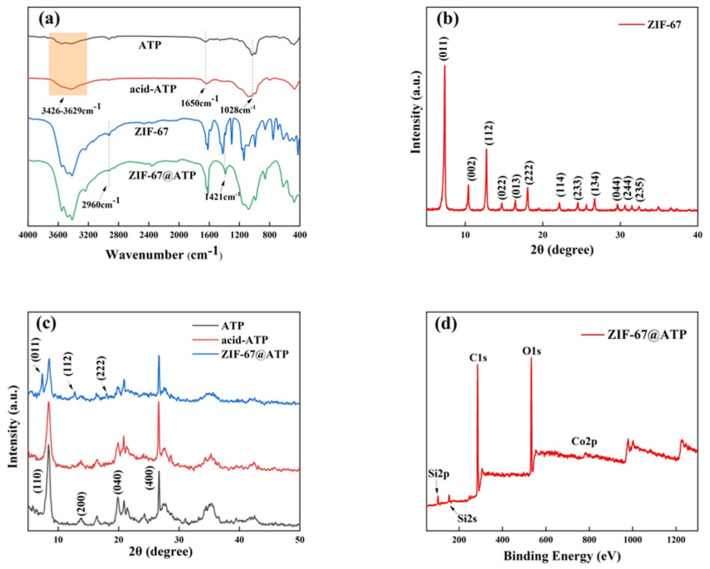
(**a**) FTIR spectra of ATP, acid-ATP, ZIF-67 and ZIF-67@ATP; (**b**) XRD patterns of the ZIF-67; (**c**) XRD patterns of ATP, acid-ATP and ZIF-67@ATP; and (**d**) XPS full spectrum of ZIF-67@ATP.

Firstly, the structure and morphology of the samples were studied. FTIR spectra of ATP, acid–ATP, ZIF-67 and ZIF- 67@ATP are shown in Figure 1a. Pure ATP has a clear absorption peak at 3426–3629 cm^−1^, which mainly represents the hydroxyl group in ATP nano porous structure [29]. The absorption peak near 1650 cm^−1^ mainly comes from the stretching vibration of zeolite water in ATP, and the absorption peak near 1028 cm^−1^ mainly corresponded to the stretching vibration of the Si-O-Si bond [30]. After acid treatment (acid–ATP sample), the original peak of ATP did not disappear, and no new peaks were observed, which showed that acid treatment has no significant effect on the chemical structure of ATP. ZIF-67 presented obvious peaks near 1139 cm^−1^ and 1303 cm^−1^ [31]. These two characteristic peaks are mainly attributed to the plane vibration of the imidazole ring. The tensile vibration absorption peak of the C=N bond is observed at 1421 cm^−1^. ZIF- 67@ATP has a new peak that appeared at 1420 cm^−1^, which corresponded to the tensile vibration peak of the C=N bond in ZIF-67. The new absorption peak at 990 cm^−1^ is related to the plane vibration of the imidazole ring in ZIF-67 [32], and a new peak appears at 2960 cm^−1^, which corresponds to the -CH_3_ tensile vibration peak in ZIF-67. All the above results indicate the successful combination of ZIF-67 and ATP.

To further characterize the structure of ZIF-67@ATP composites, XRD was recorded, and the corresponding diffractograms are presented in Figure 1b,c. The basal reflection of pure attapulgite at around 2θ = 8.2°, 13.7°, 19.8° and 26.7° shows characteristic peaks, which were assigned to the (110), (200), (040) and (400) crystal planes of attapulgite, respectively [33]. After acid treatment, the characteristic peak of attapulgite does not change, which shows that the acid treatment process has no obvious effect on the crystal structure of attapulgite. ZIF-67@ATP has three new basal reflections at around 2θ = 7.4°, 12.7°and 18.06°, respectively (Figure 1c), which were assigned to the (011), (112) and (222) crystal planes of ZIF-67, indicating that ZIF-67 is present in the modified attapulgite [34].

In addition, elements on the surface of the ZIF-67@ATP composite can be further assessed by X-ray photoelectron spectroscopy. As shown in the survey spectra in Figure 1d, corresponding to ZIF-67@ATP, main characteristic peaks are observed at 781.5 eV, 533.2 eV, 285.2 eV and 103.1 eV which correspond to Co2p, O1s, C1s and Si2p signals, respectively [35].

**Figure 2:**
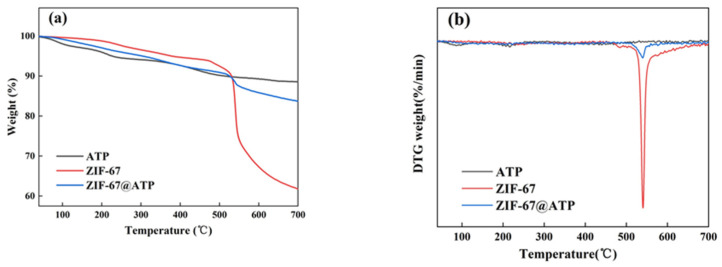
(**a**) TGA curves of ATP, ZIF-67 and ZIF-67@ATP; (**b**) DTG curves of ATP, ZIF-67 and ZIF-67@ATP.

Thermal analysis of the samples was obtained via TGA. It can be observed from Figure 2a that there was no severe weight loss of pure ATP during the whole test. The mass loss is about 12 wt%, mainly due to the loss of the adsorbed water and crystal water on the surface and internal channels of the ATP. According to the DTG (derivative thermogravimetry) presented in Figure 2b, the thermal weight loss at a temperature of less than 530 °C is mainly due to the decomposition of uncoordinated organic ligands on the surface of ZIF-67 [36]. Around 530 °C, ZIF-67 has obvious thermal decomposition behavior, which is mainly due to the destruction of ZIF-67 crystal structure. The thermal weight loss behavior of ZIF-67@ATP before 530 °C was similar to pure ATP and has the same severe weight loss as ZIF-67 at 530 °C. We also roughly estimated the load amount of ZIF-67 on ATP through TGA data, which is summarized in Table 2. It was assumed that ZIF-67@ATP contained no impurities, and the thermogravimetric behavior was the same for the unmodified ATP. Then, the mass of ATP was set as x and the mass of ZIF-67 as y, resulting in the binary first-order equations shown in Equation (1). The calculation results show that the load amount of ZIF-67 in ATP was about 18.4%.

The load amount of ZIF-67 on ATP was calculated by the following equation system:

x + y = 6.62
0.8855x + 0.6124y = 5.53(1)

**Figure 3:**
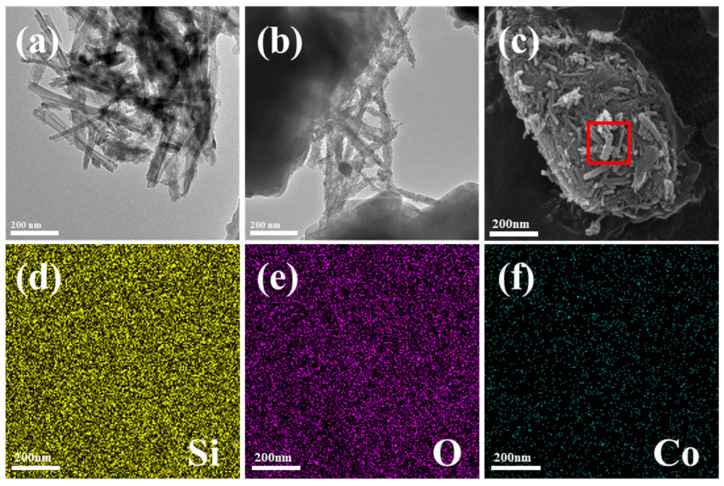
(**a**) TEM image of ATP; (**b**) TEM image of ZIF-67@ATP; (**c**) SEM image of ZIF-67@ATP; EDS mapping distribution of (**d**) Si; (**e**) O; and (**f**) Co.

Morphology of ATP and ZIF-67@ATP was studied by TEM. Figure 3a shows that the surface of unmodified ATP is smooth and an agglomeration phenomenon is clearly observed. According to Figure 3b, it was found that the ZIF-67 growing on the surface of attapulgite was significantly rougher and with reduced overlap. Therefore, the dispersion of ZIF-67 growing on the attapulgite was better than that of the original attapulgite. Figure 3c shows the SEM image of ZIF-67@ATP, and Figure 3d to Figure 3f show the EDS mapping distribution of the Si, O and Co elements of the square area in Figure 3c. As shown in Figure 3f, Co appeared on the surface of ATP after modification. The results show that ZIF-67 successfully combined with ATP and introduced the flame retardant elements into ATP.

### 3.2. Characterization of Flame Retardancy of EVA/MH/ZIF-67@ATP Composites

**Figure 4:**
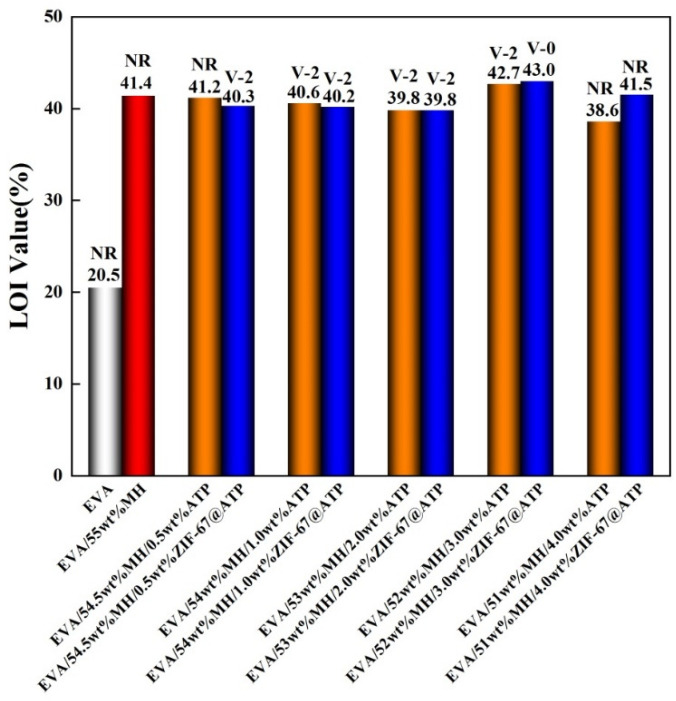
LOI and UL-94 results for EVA and EVA composites.

The main results of flame retardancy (LOI and UL-94 test) characterization are shown in Figure 4. When the content of ZIF-67@ATP reached 3 wt%, the LOI of the EVA/MH/ZIF-67@ATP composites reached 43%, which was the highest among all composites. In addition, the change in the flame retardancy properties of composites could be further analyzed by the UL-94 test. The EVA composites with 55% MH did not obtain combustion grade in UL-94. When 0.5 wt% ZIF-67@ATP was added to EVA/MH composites as a flame retardant synergist, the composite obtained V-2 grade in UL-94. However, when the addition amount of ZIF-67@ATP reached 3 wt%, the V-0 grade of the composite was obtained in UL-94. This effect might be produced by the in situ growth of ZIF-67 on the surface of ATP, which improved the dispersion of ATP and the thermal stability of the composite at the initial stage of combustion.

**Figure 5:**
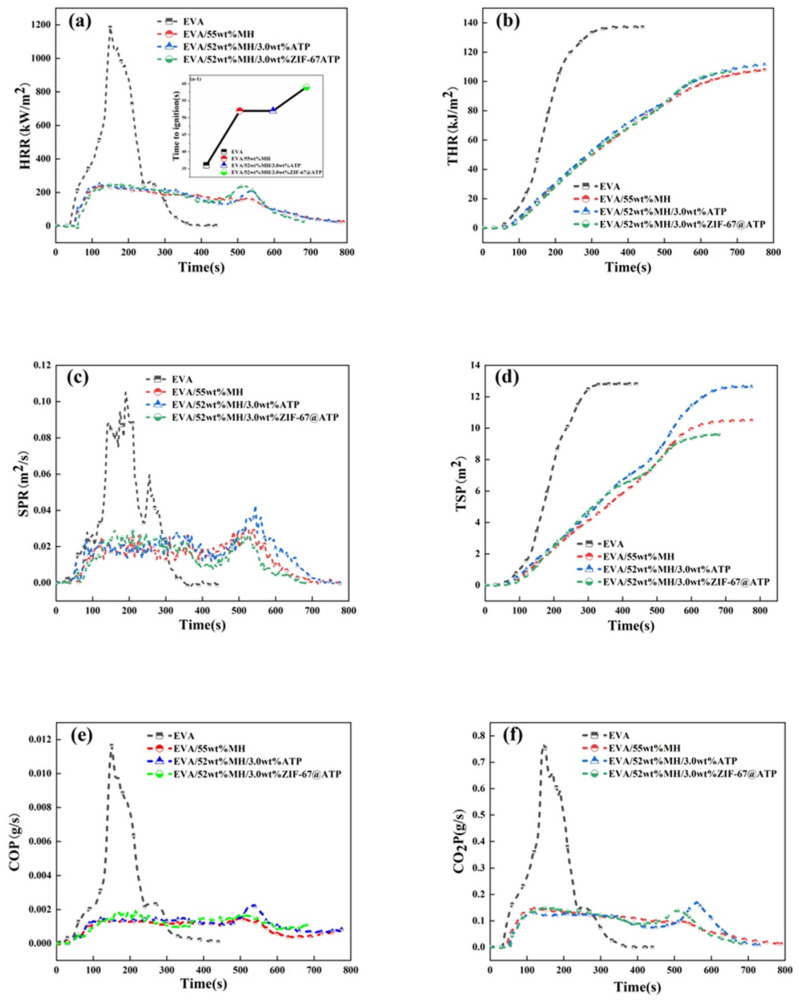
(**a**) Heat release rate (HRR); (**b**) total heat release (THR); (**c**) smoke product rate (SPR); (**d**) total smoke production (TSP); (**e**) CO production (COP); and (**f**) CO_2_ production (CO_2_P) curves of EVA, EVA/MH, EVA/52%MH/3%ATP and EVA/52%MH/3%ZIF-67@ATP samples.

Cone calorimeter test (CCT) is a kind of forced combustion test, which can reflect the flame retardancy of materials comprehensively. The cone calorimetric data are summarized in Table 3. As shown in Figure 5a, pure EVA is extremely flammable and has a sharp HRR peak that appears with the pHRR as high as 1184 kW/m^2^. After adding MH to EVA as a flame retardant, the ignition time of the composites increased from 36 s to 52 s compared with pure EVA. Moreover, there are two peaks in the heat release rate curve of EVA/MH composites. The first peak appears at 130 s, and the pHRR is 238 kW/m^2^. The second peak appears at 525 s, and the pHRR is 165 kW/m^2^. When adding 3 wt% ZIF-67@ATP, the ignition time of the composites was delayed to 59 s, and the first peak heat release rate was delayed by 45 s compared with EVA/MH composites. This means that ZIF-67@ATP can improve the thermal stability of the composite and make the composite burn violently, which needs to consume more heat [37]. The second peak may occur for two reasons. On one hand, the gas generated during combustion destroys the formed carbon layer, resulting in the contact of combustible gas and heat with the matrix materials. On the other hand, the heat is generated by the decomposition of ZIF-67. After the second peak, compared with EVA/MH, the heat release rate of EVA/MH/ZIF-67@ATP composites decreased significantly compared with EVA/MH, which may be due to the decomposition of ZIF-67 into cobalt oxides. These cobalt oxides have excellent catalytic charring performance, which can generate a dense, continuous and protective carbon layer to isolate oxygen, heat and combustible gases [38]. The combustion time of EVA/MH/ZIF-67@ATP composites increased from 440 s to nearly 700 s compared with pure EVA, which proved that the addition of ZIF-67@ATP makes the combustion behavior of EVA change from typical noncarbonized material to typical carbonized material. As shown in Figure 5e, EVA/MH/ZIF-67@ATP composites could delay the production of CO in the early stage of combustion. This phenomenon is consistent with the heat release process of composites. Finally, as shown in Figure 5f, the CO_2_ production of the composite decreased slightly compared with the EVA/MH composite after adding ZIF-67@ATP, which may be due to the catalytic charring performance of cobalt oxides [39,40].

### 3.3. Char Layer Analysis of EVA/MH/ZIF-67@ATP Composites

**Figure 6:**
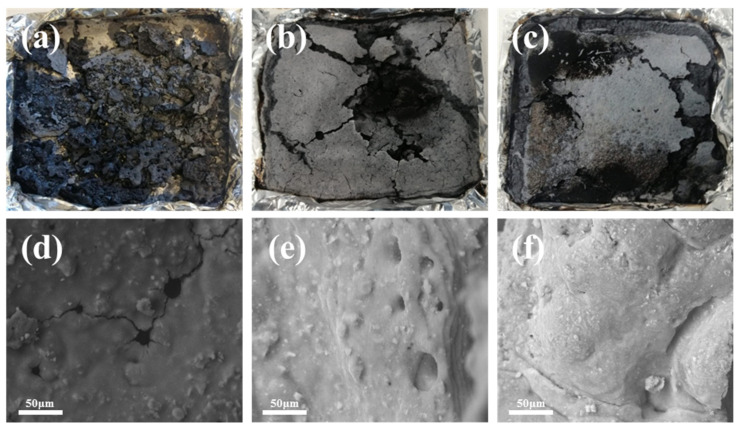
Digital images of chars from EVA composites: (**a**) EVA/55wt%MH; (**b**) EVA/52wt%-MH/3wt%ATP; (**c**) EVA/52wt%MH/3wt%ZIF-67@ATP and SEM image of chars from EVA composites; (**d**) EVA/55wt%MH; (**e**) EVA/52wt%MH/3wt%ATP; and (**f**) EVA/52wt%MH-/3wt%ZIF-67@ATP.

The research on the morphology of residual carbon can help us analyze the flame retardant synergistic mechanism of ZIF-67@ATP. The digital photos and SEM photos of EVA/55wt%MH, EVA/52wt%MH/3wt%ATP and EVA/52wt%MH/3wt%ZIF-67@ATP after the cone calorimeter test were obtained and are shown in Figure 6. It can be found that the residual carbon of the EVA/MH composite is thin and brittle, which showed that the residual carbon strength of the EVA/MH composite is relatively weak according to Figure 6a. Figure 6d shows the SEM image of the carbon residue belonging to the EVA/55wt%MH composite. Obvious holes and cracks on the carbon residue surface can be found. These cracks and holes will provide contact between oxygen and the unburned polymer materials during combustion, which leads to the accelerated overflow of combustible gas. Therefore, the combustion of the composite becomes more violent [41]. Figure 6b is a digital photo of the residual carbon of the EVA/52wt%MH/3wt%ATP composite. It can be found that the surface integrity of residual carbon was significantly improved. In Figure 6e, it can be found that the morphology of residual carbon is more continuous, and no obvious cracks were found, except for some small holes. This may be due to ATP filling the gaps generated during the combustion of the composite and further enhancing the carbon layer. According to Figure 6f, the carbon residue surface of the composite is smoother, and no holes or cracks are found in the SEM image after adding ZIF-67@ATP, which proved that ZIF-67@ATP could catalyze the formation of a strong protective carbon layer in the combustion process of polymer materials.

### 3.4. Mechanical Properties of EVA/MH/ZIF-67@ATP Composites

**Figure 7:**
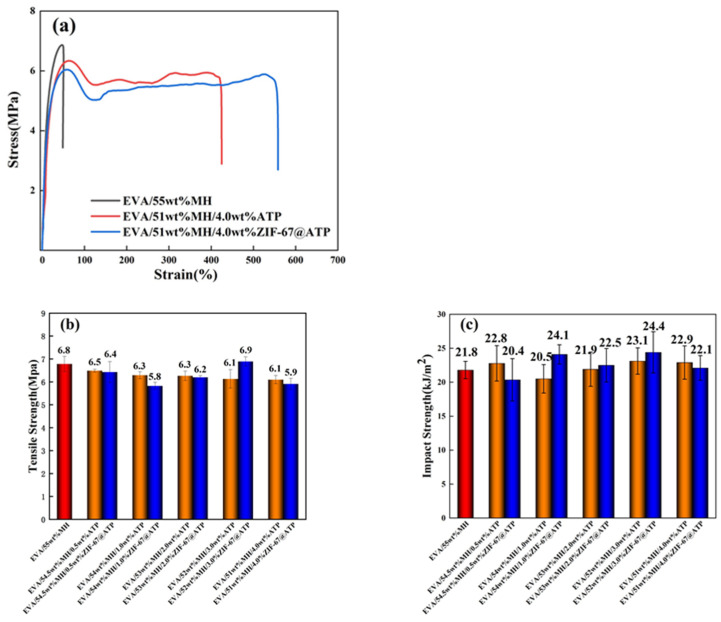
(**a**) Stress–strain curve of the EVA composites; (**b**) tensile strength; (**c**) non-notched impact strength of EVA/MH/ATP composites and EVA/MH/ZIF-67@ATP composites.

Figure 7a shows the stress–strain curves of the EVA composites, and Figure 7b shows the distribution of tensile strength of the EVA/MH/ATP composite while Table 4 shows the summary of mechanical properties data obtained. It can be found that when untreated ATP and modified ATP were added as flame retardant synergists, the tensile strength of the composites generally showed a downward trend, but the decrease in tensile strength is not obvious. Therefore, the addition of flame retardant synergists did not degrade the mechanical properties of the composites significantly. However, after adding ZIF-67@ATP as synergists into EVA/MH composites, the elongation at break of the composites increased significantly, and with the increase in the synergists, the elongation at break of the composites also increased gradually. When the addition of ZIF-67@ATP reached 4 wt%, the elongation at break of the composite increased from 69.5% to 522.2% compared with the EVA/MH composite. In addition, when the flame retardant synergist reached 4 wt%, the impact strength of the composites was greater than that of the composites without the synergist.

**Scheme 2:**
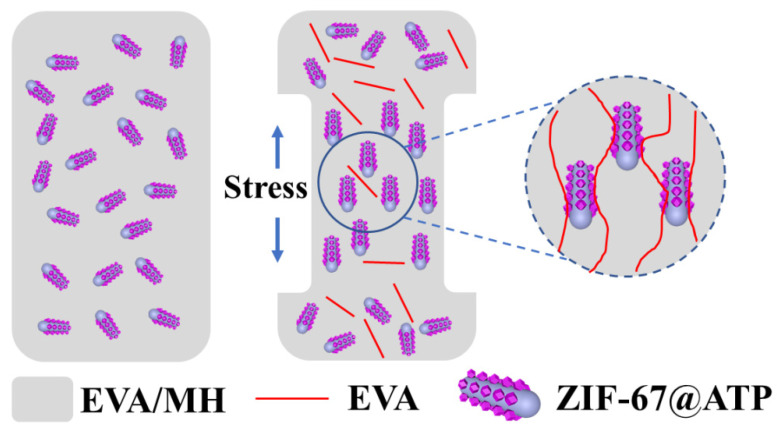
Mechanical strengthening mechanism of composites @EVA/MH.

According to the above results, the synergistic mechanism of ZIF-67@ATP in the EVA/MH composites proposed is shown in Scheme 2. The mechanical strengthening mechanism can be summarized as the following reasons: (1) ZIF-67@ATP orientation and sliding during the stress process, which transfers the stress of EVA to ZIF-67@ATP, so the mechanical properties of the composite are improved, especially the elongation at break [42]; (2) the in situ growth of ZIF-67 on the surface of ATP increases the specific surface area of ATP and improves the interaction between ZIF-67@ATP and the EVA molecular chain through hydrogen bonding [43,44]. In the tensile process of composites, the orientation and cooperative movement of ZIF-67@ATP and the corresponding crack pinning effect are the main reasons for the enhancement of the mechanical properties of composites.

## 4. Conclusions

In summary, through a solid–liquid two-phase reaction, ZIF-67 in situ grown attapulgite was successfully prepared and further blended with EVA/MH composites. The experimental results show that the addition of ZIF-67 increased the LOI of the composite to 43% and passed the V-0 grade of the UL-94 test. In the CCT experiment, it was found that the pHRR of the composites did not change significantly, and the total smoke release decreased slightly. The tensile strength and impact strength of the composites did not change significantly, but the elongation at break increased from 69.5% to 522.2% compared with EVA/MH composites. ZIF-67 can improve the thermal stability of the composites at the initial stage of combustion. The orientation and cooperative movement of ZIF-67 occur when stressed, which transfers the stress received by EVA to ZIF-67@ATP, and the elongation at break of the composite is clearly improved.

## Figures and Tables

**Table 1:** Notation and composition of EVA/MH/ZIF-67@ATP composites.

Samples	EVA/g	MH/g	ATP/g	ZIF-67@ATP/g
EVA	150	/	/	/
EVA/55wt%MH	67.5	82.5	/	/
EVA/54.5wt%MH/0.5wt%ATP	67.5	81.75	0.75	/
EVA/54wt%MH/1.0wt%ATP	67.5	81	1.5	/
EVA/53wt%MH/2.0wt%ATP	67.5	79.5	3	/
EVA/52wt%MH/3.0wt%ATP	67.5	78	4.5	/
EVA/51wt%MH/4.0wt%ATP	67.5	76.5	6	/
EVA/54.5wt%MH/0.5wt%ZIF-67@ATP	67.5	81.75	/	0.75
EVA/54wt%MH/1.0wt%ZIF-67@ATP	67.5	81	/	1.5
EVA/53wt%MH/2.0wt%ZIF-67@ATP	67.5	79.5	/	3
EVA/52wt%MH/3.0wt%ZIF-67@ATP	67.5	78	/	4.5
EVA/51wt%MH/4.0wt%ZIF-67@ATP	67.5	76.5	/	6

**Table 2:** TGA data for ATP, ZIF-67 and ZIF-67@ATP.

Samples	T_5 wt%_ (°C)	T_max_ (°C)	M_1_ (mg)	R_700_ (%)	M_2_ (mg)
ATP	227	199	6.25	88.55	5.53
ZIF-67	371	535	6.01	61.24	3.68
ZIF-67@ATP	297	534	6.62	83.46	5.53

Note: T_5 wt%_ means temperature at 5 wt% mass loss; T_max_ means temperature at the maximum degradation rate; M_1_ means sample mass before test; R_700_ means sample mass residue rate at the end of the test; M_2_ means sample mass after test.

**Table 3:** CCT data of EVA and EVA composites.

Samples	TTI (s)	pHRR (kW/m^2^)	T_pHRR_ (s)	THR (MJ/m^2^)	TSP (m^2^)	FIGRA (kW/(m^2^.s))
EVA	38 ± 3	1208 ± 34	153 ± 4	131 ± 1	12.8 ± 0.1	7.9
EVA/MH	51 ± 1	240 ± 3	130 ± 0	109 ± 2	10.0 ± 0.7	1.8
EVA/52%MH/3%ATP	54 ± 2	275 ± 36	120 ± 0	110 ± 2	12.3 ± 0.1	2.1
EVA/52%MH/3%ZIF-67@ATP	56 ± 5	268 ± 28	175 ± 0	106 ± 1	10.9 ± 1.9	1.4

**Table 4:** Mechanical properties data of EVA and EVA composites.

Samples	Tensile Strength (Mpa)	Impact Strength (kJ/m^2^)	Elongation at Break (%)
EVA/55wt%MH	6.8 ± 0.3	21.8 ± 1.3	69.5 ± 23.8
EVA/54.5wt%MH/0.5wt%ATP	6.5 ± 0.1	22.8 ± 2.6	104.6 ± 30.5
EVA/54wt%MH/1.0wt%ATP	6.3 ± 0.2	20.5 ± 2.1	160.8 ± 39.7
EVA/53wt%MH/2.0wt%ATP	6.3 ± 0.2	21.9 ± 2.5	222.4 ± 51.5
EVA/51wt%MH/4.0wt%ATP	6.1 ± 0.2	22.9 ± 2.4	444.3 ± 27.7
EVA/54.5wt%MH/0.5wt%ZIF-67@ATP	6.4 ± 0.5	20.4 ± 3.1	161.4 ± 24.1
EVA/54wt%MH/1.0wt%ZIF-67@ATP	5.8 ± 0.2	24.1 ± 1.4	118.1 ± 12.9
EVA/53wt%MH/2.0wt%ZIF-67@ATP	6.2 ± 0.1	22.5 ± 2.5	309.7 ± 0.4
EVA/51wt%MH/4.0wt%ZIF-67@ATP	5.9 ± 0.2	22.1 ± 1.8	522.2 ± 51.0

## Data Availability

Data available under reasonable request.
